# Conduction Velocity Distribution Reveals Fast-Fiber–Dominant Dysfunction in Acrylamide-Induced Peripheral Neurotoxicity

**DOI:** 10.3390/brainsci16070671

**Published:** 2026-06-26

**Authors:** Nuray Semiz, Seckin Tuncer

**Affiliations:** 1 Department of Biophysics, Faculty of Medicine, Izmir Bakircay University, 35665 Izmir, Türkiye; nuray.semiz@bakircay.edu.tr; 2 Department of Biophysics, Faculty of Medicine, Eskisehir Osmangazi University, 26040 Eskisehir, Türkiye

**Keywords:** acrylamide, conduction velocity distribution, nerve excitability, oxidative stress, peripheral neuropathy

## Abstract

**Highlights:**

**What are the main findings?**

- Acrylamide exposure caused a marked leftward shift in sciatic nerve conduction velocity distribution, with a preferential reduction in the fast-conducting fiber component.
- Fast-fiber dysfunction was accompanied by reduced nerve excitability, altered compound action potential parameters, increased oxidative stress, and histomorphometric evidence of myelin and axonal alterations.

**What are the implications of the main findings?**

- Conduction velocity distribution analysis can reveal fiber population–specific dysfunction that may be masked by conventional compound action potential or nerve conduction parameters.
- The preferential vulnerability of fast-conducting fibers provides a functional framework for understanding large-fiber involvement in acrylamide-induced peripheral neurotoxicity.

**Abstract:**

**Background:** Acrylamide (ACR) is a well-established neurotoxicant, but whether peripheral nerve dysfunction develops uniformly across all myelinated fibers or preferentially affects specific fiber subpopulations under acute high-dose exposure conditions remains unclear. This study tested the hypothesis that acute high-dose ACR exposure differentially affects fibers with distinct conduction velocities. **Methods:** Adult male Sprague-Dawley rats received daily intraperitoneal injections of ACR (50 mg/kg/day) or saline for 11 days, representing an acute high-dose experimental model of ACR-induced peripheral neurotoxicity. Sciatic nerves were assessed using compound action potential (CAP) recordings and conduction velocity distribution (CVD) analysis. Nerve excitability, oxidative stress markers, and histomorphometric parameters were also evaluated. **Results:** Acute high-dose ACR exposure induced marked body weight loss and reduced nerve excitability, reflected by increased rheobase and decreased chronaxie. CAP analysis showed significant reductions in amplitude, area, and conduction velocities. The most pronounced decrease occurred in maximum upstroke velocity, indicating preferential impairment of fast-conducting fibers. CVD analysis confirmed a marked leftward shift, with a significant reduction in fibers >50 m/s, while slower fibers were relatively preserved. These functional changes were accompanied by increased oxidative stress and supported by histomorphometric evidence of myelin and axonal alterations. **Conclusions:** In this acute high-dose rat model, ACR preferentially impaired fast-conducting, large-diameter myelinated fibers, revealing a fiber population–specific pattern of peripheral neurotoxicity. CVD analysis sensitively detected this selective fiber involvement, supported by structural evidence of myelin and axonal damage. These findings should be interpreted within the context of a short-duration, high-dose experimental paradigm rather than as a direct model of chronic low-level dietary or occupational exposure.

## 1. Introduction

Acrylamide (ACR) is an industrial chemical and a dietary contaminant formed during high-temperature cooking via the Maillard reaction, present in commonly consumed foods such as potatoes, bakery products, coffee, and plant-derived foods [1]. Human exposure is therefore widespread and largely unavoidable [2]. The International Agency for Research on Cancer has classified ACR as Group 2A (probably carcinogenic to humans), and comprehensive toxicological profiles have been established by the U.S. Environmental Protection Agency and the Agency for Toxic Substances and Disease Registry [3,4,5]. The neurotoxic potential of ACR has been extensively documented, with experimental and epidemiological studies reporting motor incoordination, skeletal muscle weakness, hindlimb dysfunction, and weight loss following exposure [6,7]. In addition to its neurotoxicity, acrylamide has been extensively characterized as a systemic toxicant affecting multiple organ systems, with cumulative dose-dependent effects described in both experimental and occupational exposure settings [8]. Following systemic absorption, ACR is metabolized to glycidamide and distributes widely across tissues, with oxidative stress emerging as a principal mechanism underlying its neurotoxicity [9,10,11].

Previous experimental and occupational studies have established that ACR exposure produces a characteristic peripheral neuropathy involving motor impairment, sensory symptoms, reduced nerve conduction, oxidative stress, and structural nerve injury. In exposed tunnel workers, Kjuus et al. [12] reported reduced ulnar sensory nerve conduction velocity and prolonged distal delay after occupational exposure to acrylamide- and N-methylolacrylamide-containing grouting agents, with partial recovery over time. A later follow-up study showed persistent subclinical effects, particularly in the sural sensory nerve, suggesting that long peripheral nerves may remain vulnerable even years after exposure [13]. Earlier experimental work in baboons similarly demonstrated that acrylamide neuropathy was accompanied by reduced action potential amplitudes, declining conduction velocities, and preferential involvement of large-diameter fibers [14]. In rodents, Zhu et al. [11] showed that ACR-induced sciatic nerve dysfunction was associated with time-dependent oxidative imbalance, including increased lipid peroxidation and reduced antioxidant capacity. More recent experimental studies have further characterized ACR neurotoxicity using behavioral, biochemical, histological, immunohistochemical, and molecular endpoints, including MBP expression, MAPK/apoptosis signaling, ERK1/2–Nrf2 signaling, PI3K/AKT signaling, and neuroinflammatory pathways [10,15,16,17]. However, these approaches largely rely on global nerve conduction indices, biochemical injury markers, or structural endpoints and therefore do not determine how different conduction velocity-defined fiber populations contribute to the observed functional impairment. Therefore, despite extensive research on ACR-induced peripheral neurotoxicity, a fundamental functional question remains unanswered. Classical nerve conduction studies measure compound action potential (CAP) amplitude, area, and mean conduction velocity parameters that represent the summed activity of thousands of axons with heterogeneous diameters and degrees of myelination. While these global parameters are useful for detecting the presence of neuropathy, they average across fiber populations and may therefore mask subpopulation-specific damage [18]. Consequently, it is not known whether ACR affects all myelinated nerve fibers uniformly or preferentially targets specific subpopulations with distinct conduction velocities. This knowledge gap has significant mechanistic and diagnostic implications, as differential vulnerability of fiber groups would point to specific pathophysiological mechanisms and could enable more sensitive detection of early neurotoxic damage. In addition, advanced electrophysiological approaches such as nerve excitability testing have been increasingly used to detect early or subclinical alterations in peripheral nerve function in toxic and metabolic neuropathies [19,20].

To overcome the limitations of conventional CAP analysis, conduction velocity distribution (CVD) methods have been developed. CVD estimates the relative contribution of different conduction velocity groups to the recorded CAP by solving an inverse problem using a mathematical model of single-fiber action potentials. This approach has been successfully applied by our group and others to characterize fiber-specific alterations in diabetic neuropathy, ischemia–reperfusion injury, and local anesthetic toxicity [18,21,22]. However, CVD analysis has not yet been applied to acrylamide-induced peripheral neurotoxicity. This represents a critical gap because conventional nerve conduction velocity (NCV) and CAP-based parameters predominantly reflect the summed behavior of myelinated fibers and cannot resolve whether the conduction deficit reflects uniform impairment across the nerve or selective functional loss of specific velocity-defined fiber populations. This distinction is particularly relevant in light of occupational and experimental evidence suggesting prominent involvement of long sensory nerves and large-diameter fibers in acrylamide neuropathy [12,13,14]. By decomposing the compound nerve response into slow-, medium-, and fast-conducting components, CVD analysis provides a functional framework to test whether ACR neurotoxicity is globally distributed or preferentially expressed in specific fiber populations.

Based on this rationale, the present study, using an acute high-dose exposure paradigm (50 mg/kg/day for 11 days), was designed to test the hypothesis that acrylamide does not uniformly impair conduction across all nerve fibers but rather differentially affects fiber populations with different conduction velocities. Specifically, we hypothesized that fast-conducting, large-diameter, thickly myelinated fibers are more vulnerable to ACR-induced damage than slow-conducting fibers due to their higher metabolic demand and greater susceptibility to oxidative stress. A secondary hypothesis was that this fiber-selective functional impairment is associated with increased oxidative stress and structural damage to axons and myelin sheaths.

The aims of the study were threefold: (1) to determine which conduction velocity groups are primarily affected by ACR neurotoxicity using CVD analysis solved via a matrix-based inverse problem with Tikhonov regularization; (2) to correlate CVD findings with morphometric parameters (axon diameter, nerve fiber diameter and g-ratio) and biochemical markers of oxidative stress (total oxidant status, total antioxidant capacity, oxidative stress index); and (3) to evaluate CVD as a sensitive, high-resolution functional biomarker for toxic peripheral neuropathy. By placing CVD-based functional mapping at the center of the analysis and integrating it with complementary structural and biochemical assessments, the present study provides a fiber population–resolved characterization of acrylamide-induced peripheral neurotoxicity.

## 2. Materials and Methods

### 2.1. Animals and Experimental Design

Experiments were performed on 20 adult (12 weeks old) male Sprague-Dawley rats weighing 350–400 g. This study was conducted in accordance with ARRIVE guidelines and EU Directive 2010/63/EU for animal experiments and approved by the KONUDAM Experimental Medicine Application and Research Center Animal Experiments Local Ethics Committee (approval number: 2021-009). Rats were housed five per cage under ambient temperature and humidity conditions on a 12/12 h light/dark cycle and fed standard rat chow and water ad libitum.

The animals were randomly divided into two groups. The acrylamide group (ACR, n = 10) received intraperitoneal (i.p.) injection of 50 mg/kg ACR (CAS: 79-06-1, Merck, Darmstadt, Germany) dissolved in saline once daily for 11 days. The control group (CON, n = 10) received a comparable volume of saline vehicle once daily for 11 days. The injection volume was adjusted daily based on body weight (approximately 0.1 mL/100 g body weight). The dose of 50 mg/kg/day was selected based on widely used experimental models of acrylamide-induced peripheral neurotoxicity, which consistently use doses in the 20–50 mg/kg range to reliably induce axonal and myelin pathology in rodents [6,23]. The highest dose within this range was chosen to ensure robust and reproducible neurotoxic effects sufficient for electrophysiological and histomorphometric characterization, as the primary aim of the study was to investigate fiber population–specific functional changes using CVD analysis rather than to establish a dose–response relationship. Although lower doses may also produce neuropathy, the 50 mg/kg/day regimen is well-established in the literature for inducing consistent and measurable peripheral nerve injury within a relatively short exposure period [10,11]. Importantly, the dose rate and cumulative exposure are critical determinants of the neurotoxic outcome [24], and the present acute high-dose paradigm should be distinguished from chronic low-dose exposure scenarios. A positive control group was not included because no standard reference compound exists for acrylamide neurotoxicity in this experimental paradigm. All injections were given daily at a fixed time.

On day 12, animals were weighed, and blood samples were collected. Rats were anesthetized with 60 mg/kg ketamine (Ketalar, Pfizer, New York, NY, USA) + 6 mg/kg xylazine (Rompun, Bayer, Leverkusen, Germany) i.p. Right and left sciatic nerves were surgically isolated as described in our previous studies [18]. The right sciatic nerve was quickly transferred into an organ bath for electrophysiological recordings. Following recording, nerves were placed vertically in 10% formaldehyde for histological examinations. Left sciatic nerves were collected in Eppendorf tubes and stored at −20 °C for biochemical analysis.

### 2.2. Electrophysiological Recordings

Electrophysiological recordings were performed using a custom-designed perfused organ bath [18]. Data acquisition was carried out with a suction electrode (inner diameter 0.5–0.8 mm) ensuring stable recording conditions. The proximal part of the dissected sciatic nerve was placed over 10 silver wires spaced 5 mm apart inside the organ bath. These silver wires were connected to the stimulator and served as stimulating electrodes. The distal part of the sciatic nerve was placed into the suction electrode.

To stimulate the sciatic nerve, an S88 stimulator (Grass, Inc., West Warwick, RI, USA) and an SIU5 stimulus isolation unit (Grass, Inc., West Warwick, RI, USA), both triggerable by the recording software (open-source CAP recording software RETICAP), were used. Recorded signals were amplified with a CP511 amplifier (Grass, Inc., West Warwick, RI, USA), digitized with a PCL-717 16-bit A/D converter (Advantech, Taipei, Taiwan), transferred to a computer, and stored on an external memory device for analysis. The nerve was continuously superfused with fresh Krebs solution at physiological temperature (36.5 ± 0.5 °C) using a peristaltic pump (Peri Star, WPI Inc., Sarasota, FL, USA) at a flow rate of 5 mL/min. The Krebs solution consisted of 119 mM NaCl, 4.8 mM KCl, 1.8 mM CaCl_2_, 1.2 mM MgSO_4_, 1.2 mM KH_2_PO_4_, 20 mM NaHCO_3_, and 10 mM glucose, with the pH adjusted to 7.4. Prior to use, the solution was equilibrated with a gas mixture of 95% O_2_ and 5% CO_2_, and aeration was maintained throughout the experimental procedures.

### 2.3. Nerve Excitability Recordings

After the nerve was prepared and stabilized for recording, rectangular pulses of 0.01–0.1 ms duration were delivered via the stimulator at a frequency not exceeding 1 Hz to determine rheobase and chronaxie values. From the data obtained under controlled conditions, a stimulus intensity–duration curve was constructed and fitted to the following equation:

$$V=\left(V_{0}-P\right)\cdot e^{\left(-\tau /t\right)}+P$$

where V_0_ is the amplitude at theoretical moment when stimulus duration is zero, *P* is the required stimulus intensity when stimulus duration is considered sufficiently long (rheobase), and τ is the constant rate of descent for the curve (chronaxie-related) [25].

Following excitability recordings, the sciatic nerve was removed from the stimulating electrodes and allowed to rest in the bath for an additional 5 min.

### 2.4. Compound Action Potential Recordings

For CAP recordings, the sciatic nerve was repositioned onto the stimulating electrodes and activated with pulses delivered by the stimulator. Supramaximal rectangular pulses of 0.1 ms duration were applied at a frequency not exceeding 1 Hz, and the resulting CAP responses were recorded. Supramaximal intensity was defined as approximately 20% above the voltage required to elicit a maximal CAP amplitude. CAPs were sampled at 40 kHz and subsequently retrieved in Microsoft Excel (Microsoft Corp., Redmond, WA, USA) for analysis.

The following parameters were derived from CAP recordings: maximum depolarization (MD, mV), defined as the maximum amplitude of the CAP; area under the CAP (mV·ms), calculated as the integral from its onset to its termination; maximum slope of the CAP rising phase (dV/dt_max_, mV/ms); and minimum slope of the falling phase (dV/dt_min_, mV/ms).

Nerve conduction velocities were calculated using two different approaches. In the first method, the time interval between the onset of the stimulus and the initiation of the CAP was measured and defined as CAP latency (L_CAP_, ms). Conduction velocity (V_CAP_, m/s) was then calculated by dividing the distance between the stimulating and recording electrodes (35 mm) by L_CAP_. In the second method, the time between stimulus onset and the point at which the CAP reached maximum depolarization was measured and defined as latency to maximum depolarization (L_MD_, ms). The distance between electrodes was divided by L_MD_, yielding a second conduction velocity parameter (V_MD_, m/s).

### 2.5. Conduction Velocity Distribution (CVD) Calculation

To obtain information about the individual activities of nerve fiber groups having different conduction velocities, CVD histograms were developed using a mathematical model that was enhanced from the model proposed by Cummins et al. [26,27] as previously described [18,28].

The basic principle of the model is based on the assumption that a recorded CAP waveform is the result of the superposition of single-fiber action potentials (SFAPs). The nerve fibers can be separated into N classes such that fibers in each class have the same SFAP waveform. The sampled CAP(t) signal can then be expressed as:

$$CAP(t)=\sum_{i=1}^{N}w_{i}\cdot f_{i}(t-\tau_{i})$$

where *N* is the number of fiber classes, *w*_i_ is the amplitude weighting coefficient for class *i*, *f_i_*(*t*) is the SFAP waveform for class *i*, and *r_i_* is the propagation delay for fibers in class *i* [18,26,27].

The weighting coefficients $w_{i}$ are general parameters that account for all influences on the contribution of each fiber class to the observed CAP, including the dependence of SFAP amplitude on conduction velocity and the number of active fibers in each class. To estimate the relative contributions of fiber groups with different conduction velocities from the recorded CAPs, an inverse problem was solved. This problem is ill-posed and requires regularization. Tikhonov regularization was applied to obtain a stable solution, with the regularization parameter *λ* determined by the L-curve method. The inverse solution was implemented using a non-negative least squares (NNLS) algorithm in MATLAB (R2023b, The MathWorks, Inc., Natick, MA, USA).

The CVD histogram was constructed for conduction velocities ranging from 10 to 90 m/s, divided into 22 bins. For ease of interpretation and to visually augment the effects of acrylamide on different nerve fiber populations, three major conduction velocity groups were defined based on the notable changes in the percentage of relative contribution: slow (<30 m/s), medium (30–50 m/s), and fast (>50 m/s) fibers. These ranges were selected based on the typical conduction velocity distribution of rat sciatic nerve myelinated fibers [18].

### 2.6. Biochemical Analysis

Blood samples collected from rats under anesthesia were allowed to clot for 30 min at room temperature, then centrifuged at 3000 rpm (750× *g*) for 10 min at +4 °C (NF 800R centrifuge, Nüve, Ankara, Turkey). Serum was collected with a micropipette, placed in Eppendorf tubes, and stored at −20 °C until analysis (within 3 months). A 3–4 cm segment of the proximal sciatic nerve was quickly dissected, placed into an Eppendorf tube, and stored at −20 °C until analysis (within 3 months). For both serum and tissue samples, all measurements were performed in a single analytical batch to minimize inter-assay variability. Nerve tissue samples were homogenized in potassium chloride buffer at a ratio of 1:9 (0.1 g tissue: 0.9 mL of 140 mM KCl) using a tissue homogenizer (Heidolph DIAX 900, Schwabach, Germany) and then centrifuged at 7000 rpm (4000× *g*) for 5 min at +4 °C. TAC and TOS measurements were performed on the supernatant. Results were not normalized to total protein concentration because the commercially available kits used (Relassay) are designed to express results as mmol Trolox equivalent/L (TAC) and μmol H_2_O_2_ equivalent/L (TOS) based on sample volume, consistent with the manufacturer’s protocol and previous studies [29].

Total antioxidant capacity (TAC) levels were measured in mmol/L using commercially available kits (Relassay, Gaziantep, Turkey) based on the bleaching of the characteristic color of the more stable ABTS (2,2′-azino-bis(3-ethylbenzothiazoline-6-sulfonic acid)) radical cation by antioxidants [29]. Data were expressed as mmol Trolox equivalent/L.

Total oxidant status (TOS) levels were measured using commercially available kits (Relassay, Gaziantep, Turkey). In this method, oxidants present in the sample oxidize the ferrous ion–o-dianisidine complex to ferric ion. The ferric ion forms a colored complex with xylenol orange in an acidic environment, and the intensity of this color, measured spectrophotometrically at 560 nm (Shimadzu UV-1800, Kyoto, Japan), reflects the total amount of oxidant molecules in the sample [29]. Results were expressed in micromoles of hydrogen peroxide equivalent per liter (μmol H_2_O_2_ equivalent/L).

To assess the redox balance, the Oxidative Stress Index (OSI, %) was calculated for both tissue and serum according to the formula [30]:

OSI=(TOS/TAC)×100

### 2.7. Histological Analysis

To evaluate pathological alterations in isolated rat sciatic nerve tissue, histological staining protocols were performed in both the CON and ACR groups. After completion of the electrophysiological recordings, segments of the right sciatic nerve were fixed in 10% neutral buffered formalin for 24 h and subsequently embedded in paraffin. Sections of 5 µm thickness were obtained for routine histological and morphometric evaluation.

For general histopathological assessment, sections were stained with hematoxylin and eosin (H&E). In a subset of formalin-fixed samples, tissues were re-fixed in 1% osmium tetroxide for 1 h before paraffin embedding and were subsequently stained with H&E to better preserve and visualize myelinated fiber profiles. Additional sections were stained with toluidine blue for evaluation of myelinated fiber density, myelin sheath morphology, and axon–myelin profiles. Osmium tetroxide-fixed sections were also stained with toluidine blue to enhance the visualization of myelinated fibers and myelin sheath morphology, as described previously [31].

Stained sections were examined under a light microscope (Olympus IX70, Tokyo, Japan) at magnifications of ×100, ×400, and ×1000. Images were captured using a digital camera (EOS650D, Canon, Tokyo, Japan). Representative low- and high-magnification images were selected to demonstrate general sciatic nerve architecture, inter-fiber spaces, myelinated fiber profiles, myelin sheath morphology, and large-diameter myelinated fibers.

Morphometric parameters, including nerve fiber diameter (µm), axon diameter (µm) and g-ratio were quantitatively assessed in both CON and ACR groups. For histopathological examination, a 3–4 mm segment from the sciatic nerve, located approximately 5–10 mm proximal to the bifurcation point where the nerve divides into the peroneal, tibial, and sural branches, was used. This segment was collected immediately after electrophysiological recordings and processed for histological analysis. For each specimen, 10 randomly selected non-overlapping fields were captured at ×400 magnification. Field selection was based on staining quality and clarity, avoiding areas with overlapping, folded, or disrupted fiber profiles. Within each field, all clearly identifiable myelinated fibers meeting predefined inclusion criteria—circular or near-circular profiles with visible axon and myelin sheath boundaries—were measured. At ×400 magnification, each field typically contained approximately 10 measurable fiber profiles, yielding a total of approximately 100 fibers per animal. Measurements were performed using image analysis software (Clemex Vision Lite v3.5, Clemex Technologies, Brossard, QC, Canada).

### 2.8. Statistical Analysis

Data obtained from experiments on isolated sciatic nerves in both experimental groups were analyzed using Microsoft Office 365 Excel. Histological parameters were quantified by performing 10 measurements per specimen on randomly selected captured images, using predefined inclusion criteria for clearly identifiable myelinated nerve fibers in each section. Measurements were expressed in micrometers (µm). All experimental procedures, including electrophysiological recordings, CAP analysis, CVD calculations, histological evaluations, and biochemical assays, were performed by investigators blinded to group allocation. Blinding was maintained throughout data collection and analysis. The distribution of variables was first assessed with the Kolmogorov–Smirnov test. For datasets conforming to normal distribution, comparisons between groups were performed with Student’s *t*-test, whereas non-normally distributed data were analyzed with the Mann–Whitney U test. For body weight analysis, between-group comparisons at each time point were performed using unpaired *t*-tests with Bonferroni correction, and within-group comparisons against Day 1 were performed using paired *t*-tests with Bonferroni correction to adjust for multiple comparisons. Correlations between variables were assessed using Pearson’s correlation coefficient for normally distributed data or Spearman’s rank correlation for non-normally distributed data. A threshold of *p* < 0.05 was considered indicative of statistical significance. All results are reported as mean ± standard error of the mean (SEM). Statistical analyses were performed using GraphPad Prism 10.0 Demo (GraphPad Software, Chicago, IL, USA).

## 3. Results

According to the experimental protocol, changes in body weight induced by the 11-day injection regimen were monitored daily in both the CON and ACR groups. For this purpose, each animal in both groups was weighed every day, and their body weights were recorded. On day nine of the experiment, one rat in the ACR group died and was excluded from all subsequent analyses. The procedures were subsequently continued with the remaining nine animals in this group (ACR, n = 9). Daily body weight measurements are presented in Figure 1 as mean ± SEM for both groups over the 11-day experimental period. Between-group comparisons at each time point were performed using unpaired *t*-tests with Bonferroni correction, and within-group comparisons against Day 1 were performed using paired *t*-tests with Bonferroni correction. Body weights did not differ between groups during the first 4 days. From day 5 onward, the ACR group exhibited significantly lower body weights compared with the CON group (*p* < 0.05), with progressive divergence until the end of the experiment. By day 11, the CON group showed a 5.0% increase in body weight (from 401.1 ± 11.7 g to 421.0 ± 6.2 g), whereas the ACR group showed an 20.3% reduction (from 404.5 ± 11.6 g to 322.3 ± 13.9 g), resulting in a statistically significant difference between groups (*p* < 0.05).

### 3.1. Acrylamide Increases Rheobase and Decreases Chronaxie

The stimulus strength–duration curve derived from the measurements provides important insights into nerve excitability. To elucidate the impact of acrylamide neurotoxicity on sciatic nerve excitability, Figure 2 presents the strength–duration curves for the CON (n = 10) and ACR (n = 9) groups plotted on the same axes (A), along with the graphs of the calculated rheobase (B) and chronaxie (C) values for both groups presented together.

Analysis of rheobase values revealed a 3.31% increase in the ACR group (2.27 ± 0.03 V) relative to the CON group (2.20 ± 0.01 V), a difference that reached statistical significance (*p* < 0.05). In contrast, chronaxie values, another key indicator of nerve excitability, were reduced by 4.54% in the ACR group (14.00 ± 0.32 µs) compared with the CON group (14.99 ± 0.28 µs). This reduction was statistically significant, with a *p* value of 0.009 (*p* < 0.05).

### 3.2. Acrylamide Reduces CAP Amplitude, Area, and Upstroke Velocity

Acrylamide administration induced distinct alterations in the CAP waveform. To illustrate these differences, representative CAP recordings that most accurately reflect the changes observed in the mean values are presented for each group in Figure 3.

Based on the recorded CAPs, maximum depolarization (MD) values and the areas under the CAP curves were calculated for both experimental groups, and the group means are presented in Figure 4.

Analysis of CAP recordings revealed a significant 37.88% reduction in MD values in the ACR group (23.61 ± 1.61 mV) compared with the CON group (38.01 ± 2.29 mV; *p* < 0.05). Similarly, the area under the CAP curve was reduced by 17.78% in the ACR group (12.75 ± 0.84 mV·ms) relative to the CON group (14.96 ± 0.54 mV·ms; *p* < 0.05).

Among the parameters calculated from the recorded CAP data, nerve conduction velocities were determined in two different ways. The mean V_CAP_ and V_MD_ values calculated for the groups are shown in Figure 5. Analysis of conduction velocity parameters revealed a 13.60% reduction in V_CAP_ values in the ACR group (78.67 ± 2.61 m/s) compared with the CON group (91.06 ± 1.94 m/s; *p* < 0.05). Similarly, V_MD_ values were reduced by 17.81% in the ACR group (48.89 ± 1.93 m/s) relative to the CON group (59.49 ± 1.39 m/s; *p* < 0.05).

Further evaluation of CAP derivatives showed marked alterations in both the rising and falling phases. The mean dV/dtmax value was decreased by 57.78% in the ACR group (104.79 ± 9.75 mV/ms) compared with the CON group (248.25 ± 17.96 mV/ms; *p* < 0.05). The mean dV/dtmin values were also significantly reduced, being 38.14% lower in the ACR group (−97.29 ± 7.62 mV/ms) than in the CON group (−157.30 ± 11.06 mV/ms; *p* < 0.05).

### 3.3. Acrylamide Selectively Reduces Fast-Conducting Fiber Contribution

To determine whether acute high-dose acrylamide exposure differentially affects nerve fiber subpopulations with distinct conduction velocities, CVD histograms were estimated from recorded CAPs using the inverse mathematical model described in the Methods section. The estimated CVD histograms for CON and ACR groups are presented in Figure 6B as smoothed curves showing the percent relative contribution of fibers across conduction velocities ranging from approximately 11 m/s to 86 m/s.

In the CON group, the CVD histogram exhibited a broad distribution with a prominent peak at higher conduction velocities. The relative contribution of nerve fibers was distributed across a wide range, with substantial contributions from fast-conducting fibers above 60 m/s. Specifically, a distinct peak was observed around 60–65 m/s, indicating a robust population of large-diameter, thickly myelinated fibers.

In contrast, the ACR group demonstrated a marked leftward shift in the CVD curve, indicating a reduction in the contribution of fast-conducting fibers. The peak of the distribution shifted from approximately 63 m/s in the CON group to approximately 48 m/s in the ACR group. Notably, the relative contribution of fibers conducting above 60 m/s was dramatically reduced in the ACR group, while the contribution of fibers conducting between 30 and 50 m/s appeared relatively preserved or slightly increased (Figure 6A).

For quantitative analysis and ease of interpretation, three major conduction velocity subgroups were defined based on the notable changes in the percentage of relative contribution: slow (<30 m/s), medium (30–50 m/s), and fast (>50 m/s) fibers. These velocity ranges were selected based on the typical conduction velocity distribution of rat sciatic nerve myelinated fibers and the observed alterations following ACR exposure. The relative contributions of these subgroups were recalculated and are presented in Figure 6A.

Analysis of the velocity subgroups revealed that acrylamide exposure induced a significant reduction in the contribution of fast-conducting fibers (>50 m/s). The relative contribution of fast fibers decreased from 0.80 ± 0.04 in the CON group to 0.52 ± 0.03 in the ACR group, representing a 35% reduction (*p* < 0.05). In contrast, the contribution of slow fibers (<30 m/s) showed a modest increase from 0.18 ± 0.02 in the CON group to 0.24 ± 0.03 in the ACR group, although this difference did not reach statistical significance (*p* > 0.05). The contribution of medium fibers (30–50 m/s) remained relatively unchanged (CON: 0.48 ± 0.03; ACR: 0.52 ± 0.04; *p* > 0.05).

### 3.4. Acrylamide Induces Systemic and Neural Oxidative Stress

Blood samples obtained under anesthesia and left sciatic nerve tissues were analyzed to determine oxidative stress markers (Table 1). In tissue samples, TOS levels in the ACR group (17.36 ± 1.88 μmol H_2_O_2_ equivalent/L) were significantly higher than those in the CON group (11.51 ± 0.46 μmol H_2_O_2_ equivalent/L), representing a 50.83% increase (*p* < 0.05). Consistently, serum analysis revealed that TOS levels in the ACR group (7.58 ± 1.17 μmol H_2_O_2_ equivalent/L) were markedly elevated by 58.25% compared with the CON group (4.79 ± 0.47 μmol H_2_O_2_ equivalent/L) (*p* < 0.05). These findings clearly demonstrate that acrylamide administration induced a pronounced systemic and tissue-specific oxidative burden.

In tissue samples, mean TAC levels in the ACR group (0.31 ± 0.12 mmol Trolox equivalent/L) were significantly decreased by 50.79% compared with the CON group (0.63 ± 0.05 mmol Trolox equivalent/L) (*p* < 0.05). A similar trend was observed in serum, where TAC levels in the ACR group (1.31 ± 0.13 mmol Trolox equivalent/L) showed a significant 14.38% reduction relative to the CON group (1.53 ± 0.05 mmol Trolox equivalent/L) (*p* < 0.05). Evaluation of OSI values, a key indicator of the balance between oxidative stress and antioxidant defense, demonstrated a marked disruption of redox homeostasis. In tissue samples, OSI levels in the ACR group (5668.12 ± 669.15) were significantly elevated by 194.34% compared with the CON group (1925.72 ± 177.29) (*p* < 0.05). Consistently, serum OSI levels in the ACR group (588.11 ± 81.22) exhibited a significant 86.45% increase relative to the CON group (315.42 ± 33.15) (*p* < 0.05). These findings clearly indicate that acrylamide exposure profoundly impairs antioxidant capacity and shifts the oxidant–antioxidant balance toward a state of pronounced oxidative stress in both tissue and systemic compartments.

### 3.5. Acrylamide Alters Myelin and Axonal Morphology

Histological evaluation of sciatic nerve sections was performed using H&E, osmium tetroxide-fixed H&E, toluidine blue, and osmium tetroxide-fixed toluidine blue staining. Representative images are presented in Figure 7 and Figure 8.

In H&E-stained sections, the CON group showed relatively preserved sciatic nerve architecture, with compactly arranged nerve fibers and regular connective tissue organization. In contrast, the ACR group showed less compact tissue organization, enlarged inter-fiber spaces, and irregular myelinated fiber profiles. These changes were also visible in high-magnification H&E images, where ACR-treated nerves showed swollen and variably shaped fiber profiles. Osmium tetroxide-fixed H&E sections provided additional visualization of myelinated fiber profiles and supported the presence of reduced fiber compactness and increased inter-fiber spacing in the ACR group compared with the CON group (Figure 7).

Toluidine blue-stained sections showed dense and relatively uniform myelinated fiber profiles in the CON group. Following ACR exposure, myelinated fiber profiles appeared less densely packed, inter-fiber spaces were more prominent, and fiber profiles appeared more heterogeneous. High-magnification toluidine blue images showed irregular myelin sheath profiles and qualitatively fewer large-diameter myelinated fibers in the ACR group. Osmium tetroxide-fixed toluidine blue sections further supported the presence of altered myelin sheath morphology in the ACR group (Figure 8).

To quantitatively assess these structural alterations, morphometric parameters including nerve fiber diameter, axon diameter and g-ratio were measured in the CON and ACR groups. The results are presented in Figure 9. Morphometric analysis revealed a significant reduction in nerve fiber diameter, which decreased by 15.63% in the ACR group (9.34 ± 0.24 µm) compared with the CON group (11.07 ± 0.14 µm; *p* < 0.05). Similarly, axon diameter was also significantly decreased by 10.77% in the ACR group (5.58 ± 0.09 µm) compared with the CON group (6.26 ± 0.08 µm; *p* < 0.05). The G-ratio (axon diameter/fiber diameter) was significantly increased in the ACR group (0.60 ± 0.01) compared with the CON group (0.57 ± 0.01; *p* < 0.05), indicating disproportionate myelin thinning relative to axonal atrophy.

In order to further explore the relationships between functional, structural, and biochemical alterations, correlation analyses were performed. Fast fiber contribution (from CVD analysis) showed a strong positive correlation with g-ratio (r = 0.79, *p* < 0.001) and a strong negative correlation with tissue OSI (r = −0.78, *p* < 0.001). CAP amplitude (MD) also showed a significant negative correlation with tissue OSI (r = −0.69, *p* < 0.01). In addition, body weight change (%) was positively correlated with fast fiber contribution (r = 0.71, *p* < 0.01) and negatively correlated with tissue OSI (r = −0.74, *p* < 0.01). These correlations support the mechanistic link between oxidative stress, structural damage, and functional impairment of fast-conducting fibers.

## 4. Discussion

Acrylamide (ACR) is a well-recognized environmental neurotoxin that induces distal axonal degeneration and peripheral neuropathy in both experimental animals and humans [6,7]. The acrylamide dosing regimen used in the present study (50 mg/kg/day) was selected based on widely employed experimental models of acrylamide-induced peripheral neurotoxicity, which consistently use doses in the 20–50 mg/kg range to reliably induce axonal and myelin pathology in rodents [6,10,23]. Consistent with previous reports, ACR-treated animals in our study exhibited progressive motor impairments and systemic manifestations including hindlimb dysfunction, muscle atrophy, and organ discoloration. It should be noted, however, that motor impairments and organ findings were not quantitatively scored. Daily body weight measurements (Figure 1) confirmed progressive weight loss in the ACR group from day 8 onward, with an 18.28% reduction by day 11 compared with a 6.5% increase in the CON group, confirming the effectiveness of the dosing regimen. The present study provides an integrated assessment of ACR-induced peripheral neurotoxicity by combining nerve excitability, conduction analyses, oxidative stress markers, and histomorphometric evaluations. Our findings demonstrate that ACR does not affect all myelinated fibers uniformly but rather selectively targets fast-conducting, large-diameter fibers, while slow-conducting fibers are relatively spared. This selective vulnerability was evidenced by multiple independent measures: a leftward shift in the conduction velocity distribution (CVD) curve, a reduction in CAP amplitude, area, dV/dt_max_ (which specifically reflects the fastest fibers), and in myelin thickness. These functional and structural alterations were underpinned by a marked increase in oxidative stress, with increased tissue OSI.

The changes observed in nerve excitability provide important context for interpreting the conduction abnormalities. The increase in rheobase indicates that axons required greater stimulus intensity to be activated, consistent with reduced membrane excitability. Although chronaxie decreased, the concurrent increase in rheobase suggests that these changes do not reflect a simple enhancement of excitability, but rather alterations in membrane properties, likely involving voltage-gated sodium channel function at the nodes of Ranvier [32]. Similar patterns of altered excitability have been described in other toxic and metabolic neuropathies, where fast-conducting fibers appear particularly vulnerable [18,33].

The CAP findings further support a non-uniform pattern of functional impairment. The reduction in overall CAP amplitude and area indicates a loss of effective fiber contribution, while the disproportionate decrease in the rising phase of the CAP suggests that the earliest and fastest-conducting components are preferentially affected. This interpretation is reinforced by the marked reduction in dV/dt_max_, a parameter dominated by the fastest fibers, whereas changes in dV/dt_min_ were comparatively less pronounced. Together, these findings point toward a selective disruption of fast-conducting fibers rather than a uniform decline across the entire fiber population.

CVD analysis provided direct evidence for this fiber population–specific effect. While additional histologically derived fiber size distribution analysis would have been informative, the CVD method used in the present study provides a validated quantitative functional estimate of fiber population composition that corresponds to structural fiber size distribution [18,28]. In control nerves, the distribution was dominated by a prominent high-velocity component corresponding to large-diameter, heavily myelinated fibers. Following ACR exposure, this high-velocity peak was markedly attenuated and shifted toward lower conduction velocities, indicating a redistribution of functional contributions within the nerve. Importantly, while the relative contribution of fast-conducting fibers was clearly reduced, the contributions of medium and slow fibers remained relatively stable. This apparent discrepancy likely reflects redistribution within the overall fiber population rather than true preservation of specific subgroups. These findings demonstrate that ACR-induced neurotoxicity manifests as a selective suppression of fast-conducting fibers rather than a global loss of conduction.

This fiber population–resolved interpretation extends previous experimental and translational observations. Earlier animal studies showed that acrylamide exposure reduces conventional conduction velocity and action potential amplitude, and the baboon model suggested that the fall in maximal conduction velocity was likely related to selective loss or dysfunction of large-diameter fibers [14]. Occupational studies in tunnel workers similarly demonstrated subclinical peripheral nerve involvement after exposure to acrylamide-containing grouting agents, including reduced sensory conduction velocity and amplitude, with the sural nerve showing delayed or persistent abnormalities [12,13]. These findings are clinically important because conventional NCV mainly samples the fastest conducting large myelinated fibers, whereas smaller or slower fiber populations contribute less directly to standard maximal conduction velocity measurements. The present study builds on this literature by using CVD analysis to resolve the relative contribution of conduction velocity-defined fiber populations within the sciatic nerve. In this way, our findings provide functional evidence that the ACR-induced conduction deficit is dominated by suppression of the fast-conducting component rather than by an equivalent reduction across all fiber populations.

The structural data provide supportive morphological context for these functional alterations. H&E and osmium tetroxide-fixed H&E sections showed less compact sciatic nerve architecture, increased inter-fiber spaces, and irregular myelinated fiber profiles following ACR exposure. Toluidine blue and osmium tetroxide-fixed toluidine blue sections further suggested altered myelin sheath morphology and qualitatively fewer large-diameter myelinated fibers following ACR exposure. It is important to note that the presence of focal axonal swelling observed in histological sections does not contradict the overall reduction in mean axon diameter measured morphometrically. Axonal swelling represents a focal pathological feature, whereas axonal atrophy reflects more generalized axonal damage. Both features have been described in acrylamide neuropathy and are not mutually exclusive [7,34]. These observations are in line with classical and more recent descriptions of acrylamide-induced neuropathy, including distal axonal degeneration, altered myelinated fiber morphology, and nerve fiber regeneration following toxic peripheral nerve injury [7,34,35,36,37]. They are also consistent with recent experimental studies reporting ACR-related sciatic nerve degeneration, reduced MBP expression, and altered myelin-associated molecular pathways [15,16,17]. Importantly, in the present study these qualitative observations were supported by morphometric analysis, which showed significant reductions in nerve fiber diameter and axonal diameter, as well as a significant increase in the G-ratio (axon diameter/fiber diameter) in the ACR group (Figure 9). The increased G-ratio indicates disproportionate myelin thinning relative to axonal atrophy. The present study extends these structural observations by linking them to a velocity-resolved functional phenotype. Given the strong dependence of conduction velocity on both axonal diameter and myelin integrity, these structural changes provide a plausible explanation for the leftward shift in conduction velocity distribution and the preferential reduction in the fast-conducting component. Thus, rather than serving as standalone neuropathological endpoints, the histological and morphometric findings complement the electrophysiological data and support the interpretation that ACR-related myelin and axonal alterations contribute disproportionately to dysfunction of large, fast-conducting myelinated fibers.

In addition to these structural alterations, the biochemical findings suggest that oxidative stress may contribute to the selective vulnerability of fast-conducting fibers. ACR exposure resulted in a marked shift in redox balance toward oxidative stress, characterized by increased oxidant load and reduced antioxidant capacity. This is consistent with Zhu et al. [11], who reported time-dependent increases in lipid peroxidation and decreases in antioxidant defense in rat sciatic nerve, with strong correlations between oxidative indices and conventional electrophysiological parameters. Similar oxidative stress-related mechanisms have also been emphasized in more recent ACR models, including studies showing altered MDA, GSH, SOD, MBP, MAPK/apoptosis signaling, ERK1/2–Nrf2 signaling, and PI3K/AKT-related protective pathways [15,16,17]. The present study does not merely confirm oxidative stress as a general mechanism of ACR neurotoxicity; rather, it links this biochemical injury pattern to a velocity-defined functional phenotype, namely preferential impairment of the fast-conducting component. Fast-conducting fibers, due to their larger size, higher metabolic activity, and greater reliance on ion channel function, may be particularly sensitive to oxidative modification of ion channels, mitochondrial dysfunction, and impaired axonal transport [37]. However, while the present findings support a relationship between oxidative stress and selective fiber vulnerability, they do not establish a direct causal mechanism.

The selective involvement of fast-conducting fibers has important translational implications. Large-diameter myelinated fibers are responsible for proprioception, vibration sense, and motor control, and their early dysfunction is a hallmark of several clinical neuropathies, including chemotherapy-induced and diabetic neuropathy [38,39]. Human occupational studies further support the relevance of large-fiber involvement in acrylamide-related neuropathy. In tunnel workers exposed to acrylamide- and N-methylolacrylamide-containing grouting agents, Kjuus et al. [12] reported reduced sensory nerve conduction velocity, prolonged distal delay, and reduced sensory amplitude, with most abnormalities being slight, subclinical, and partially reversible over time. A later follow-up study showed persistent subclinical abnormalities, particularly reduced sural sensory nerve conduction velocity, suggesting that long peripheral sensory nerves may remain vulnerable even years after exposure [13]. Experimental primate data also support this interpretation: Hopkins and Gilliatt [14] showed that acrylamide neuropathy in baboons was accompanied by reduced action potential amplitudes, declining conduction velocities, and preferential involvement of large-diameter fibers. The present findings are consistent with these translational observations but extend them by showing, in a controlled experimental setting, that the functional impairment can be resolved into a preferential reduction in the fast-conducting fiber contribution. Thus, ACR exposure may not simply reduce global nerve output; rather, it appears to disproportionately affect the functional component most closely associated with large, rapidly conducting myelinated fibers. Moreover, the ability of CVD analysis to resolve redistribution of fiber contributions highlights its potential as a sensitive experimental tool for detecting early or subclinical toxic neuropathy before severe abnormalities become evident in conventional conduction parameters.

Several limitations of this study should be acknowledged. Only a single dose of acrylamide (50 mg/kg/day) was tested. This dose was selected based on established literature demonstrating reliable induction of peripheral neuropathy [23]; however, dose–response studies are needed to determine whether similar fiber-selective effects occur at lower exposure levels. Although the selected dose does not directly reflect typical human exposure and the high-dose, short-duration i.p. paradigm should not be considered a direct model of dietary or occupational exposure, it allowed robust investigation of the underlying mechanisms of fiber-specific vulnerability. Therefore, the present findings should be interpreted primarily in a mechanistic context. Furthermore, the study used only an acute high-dose exposure paradigm; whether similar fiber-selective effects occur under chronic low-dose exposure or whether the observed alterations are reversible after exposure cessation remains to be determined. In addition, only male rats were included; sex-dependent differences in acrylamide metabolism and neurotoxicity have been reported, and future studies should include female animals. Furthermore, no ultrastructural evaluation (electron microscopy) or immunohistochemical analyses were performed. Although such approaches could provide additional mechanistic insights, they were beyond the scope of the present study, which was designed to characterize functional (electrophysiological and CVD-based) alterations and their correlation with biochemical and histomorphometric changes. One animal in the ACR group died before the end of the experiment and was excluded from all analyses. While such mortality is commonly observed in high-dose acrylamide studies and may reflect severe systemic toxicity, the exclusion could theoretically introduce survivorship bias; however, the remaining ACR animals displayed consistent and statistically significant neurotoxic alterations across multiple independent measures, supporting the robustness of the findings. In addition, motor impairments and organ discoloration were recorded only as macroscopic observations without quantitative scoring. Histological observations were used primarily as supportive structural evidence rather than as standalone neuropathological endpoints, and should be interpreted together with the quantitative morphometric measurements and CVD-based functional data.

## 5. Conclusions

In conclusion, this study demonstrates that acrylamide preferentially impairs fast-conducting, large-diameter myelinated fibers in the rat sciatic nerve, while slower-conducting fibers are relatively less affected. This selective toxicity was primarily evidenced by CVD analysis and supported by convergent findings from nerve excitability parameters, CAP parameters, oxidative stress markers, and histomorphometric reductions in myelin thickness, nerve fiber diameter, and axon diameter. The close correspondence between the magnitude of fast fiber loss, myelin thinning, and oxidative stress elevation supports a mechanistic model in which ACR-induced oxidative damage preferentially affects the most metabolically demanding fiber populations. Importantly, these findings establish CVD analysis as a high-resolution, fiber population–sensitive tool that reveals a previously unresolved feature of ACR neurotoxicity: preferential functional suppression of the fast-conducting component of the sciatic nerve. Future studies should investigate the molecular mechanisms underlying this selective vulnerability, explore whether antioxidant interventions can protect fast fibers from ACR-induced injury, and evaluate the translational potential of CVD analysis for early detection of subclinical neuropathy in at-risk populations.

## Figures and Tables

**Figure 1:**
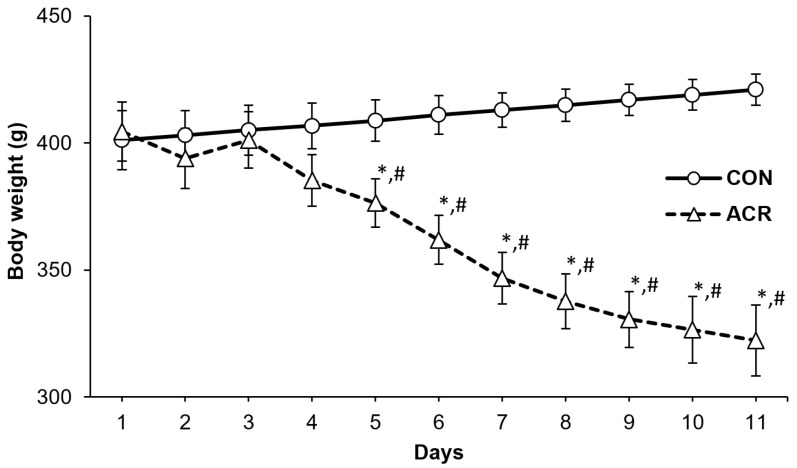
Effects of acrylamide exposure on body weight during the 11-day experimental period. Body weight was recorded daily in the CON (n = 10) and ACR (n = 9) groups. Data are presented as mean ± SEM. * *p* < 0.05, vs. CON at the same time point (unpaired *t*-test with Bonferroni correction for multiple comparisons). # *p* < 0.05, vs. Day 1 within the same group (paired *t*-test with Bonferroni correction).

**Figure 2:**
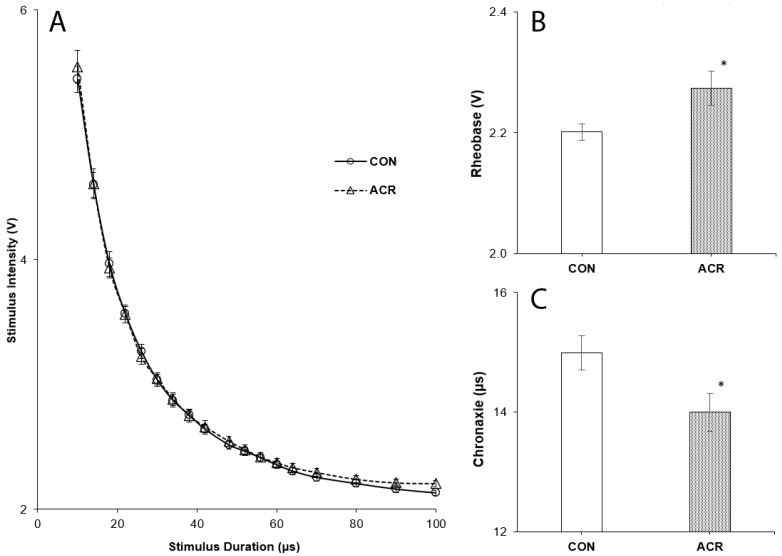
Stimulus intensity (V) vs. duration (µs) data with fitted curves (nonlinear least-squares regression) (**A**), calculated Rheobase (**B**) and Chronaxie (**C**) values from sciatic nerves of CON (n = 10) and ACR (n = 9) groups. Data are expressed as mean ± SEM. * indicates a statistically significant difference between groups (*p* < 0.05).

**Figure 3:**
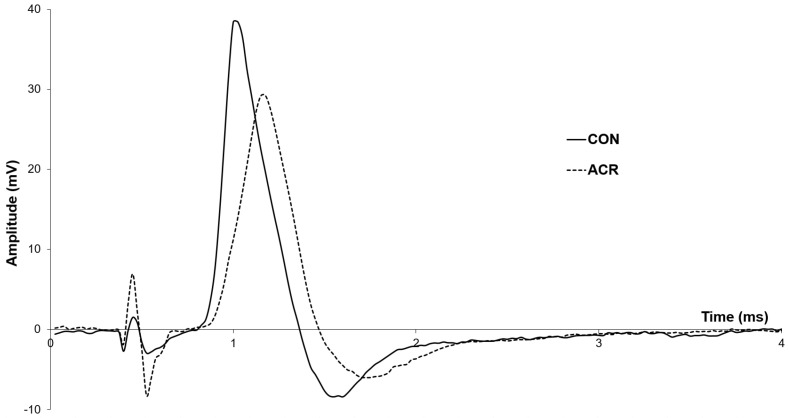
Representative compound action potential (CAP) recordings illustrating alterations in waveform patterns. Traces were selected as the recordings that most closely matched the group mean values for key CAP parameters (maximum depolarization, area, and conduction velocity), thereby providing a visual representation of the typical response for each group.

**Figure 4:**
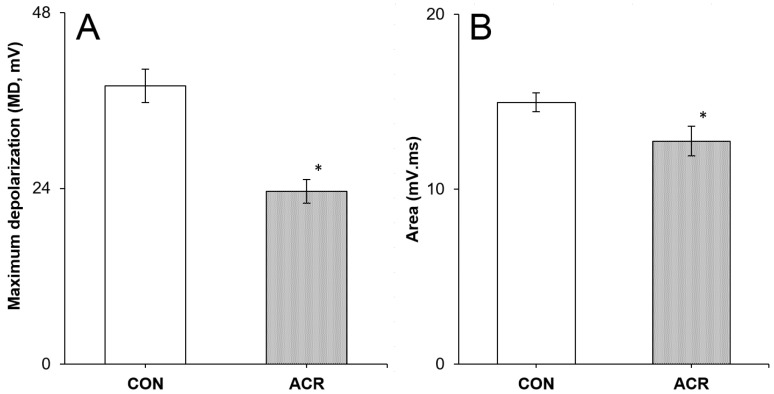
Maximum Depolarization (MD, mV) (**A**) and Area (mV·ms) (**B**) values of CAPs recorded from sciatic nerves of CON (n = 10) and ACR (n = 9) groups. Data are expressed as mean ± SEM. * indicates a statistically significant difference between groups (*p* < 0.05).

**Figure 5:**
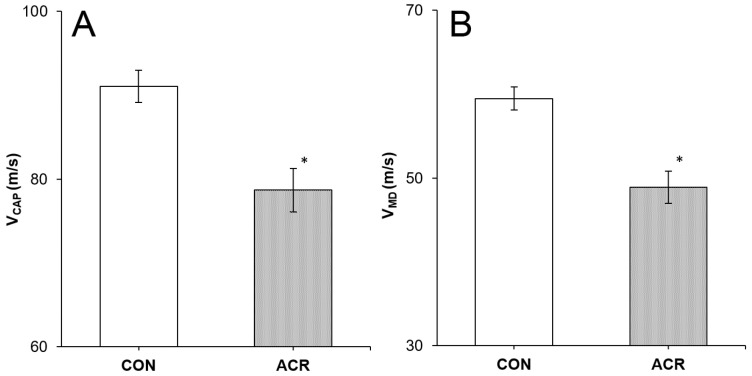
Measured conduction velocity values, V_CAP_ (**A**) and V_MD_ (**B**) of CAPs recorded from sciatic nerves of CON (n = 10) and ACR (n = 9) groups. Data are expressed as mean ± SEM. * indicates a statistically significant difference between groups (*p* < 0.05).

**Figure 6:**
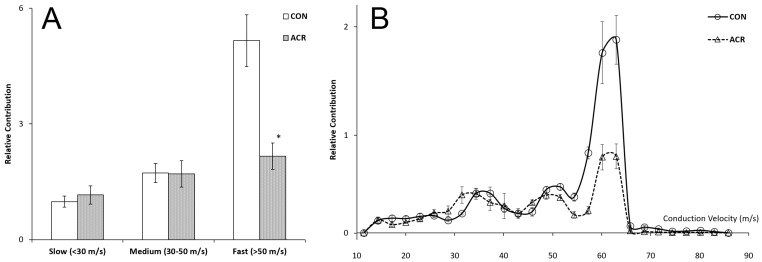
Conduction velocity distribution (CVD) analysis demonstrating fiber population–specific alterations in rat sciatic nerve following acrylamide exposure. (**A**) Smoothed CVD curves showing the percent relative contribution of nerve fibers across conduction velocities (m/s) in control (CON, n = 10) and acrylamide-treated (ACR, n = 9) groups. Note the leftward shift and reduced contribution of fast-conducting fibers (>60 m/s) in the ACR group. (**B**) Quantitative analysis of relative contributions of slow (<30 m/s), medium (30–50 m/s), and fast (>50 m/s) conducting fibers. Data are presented as mean ± SEM. * *p* < 0.05 vs. CON group.

**Figure 7:**
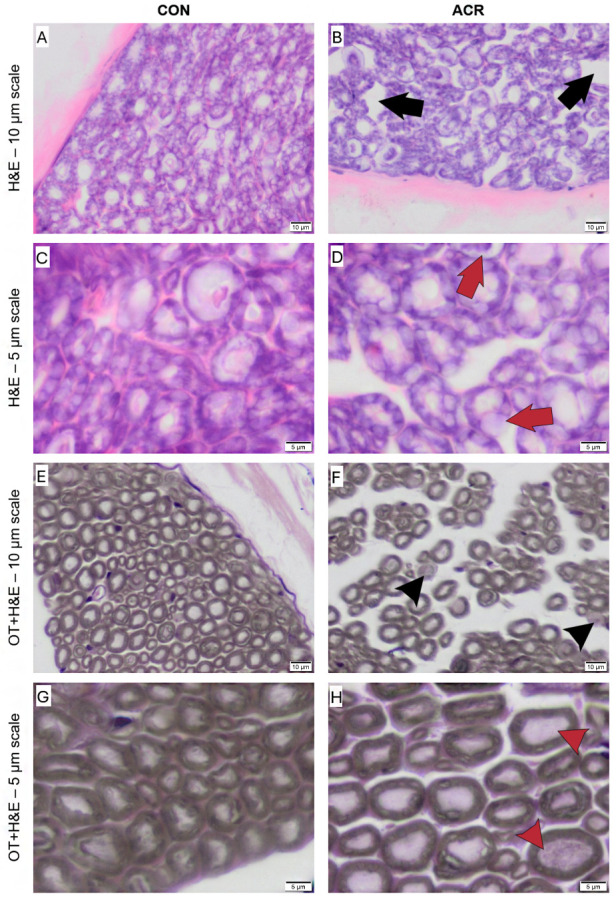
Representative H&E and osmium tetroxide-fixed H&E sections of rat sciatic nerve from the control (CON) and acrylamide-treated (ACR) groups. Panels (**A**–**D**) show H&E-stained sections, and panels (**E**–**H**) show osmium tetroxide-fixed H&E sections. Low-magnification images are shown in panels (**A**,**B**,**E**,**F**), whereas high-magnification images are shown in panels (**C**,**D**,**G**,**H**). Compared with the CON group, ACR exposure disrupted the overall sciatic nerve architecture, increased inter-fiber spaces, and caused irregularity and disorganization of myelinated fiber profiles. Arrows indicate representative pathological features: black arrows in (**B**) indicate endoneurial edema; red arrows in (**D**) indicate myelin degeneration; black arrowheads in (**F**) indicate neurofilamentous axonal swelling; red arrowheads in (**H**) indicate axonal swelling. Scale bars: 10 µm in (**A**,**B**,**E**,**F**); 5 µm in (**C**,**D**,**G**,**H**).

**Figure 8:**
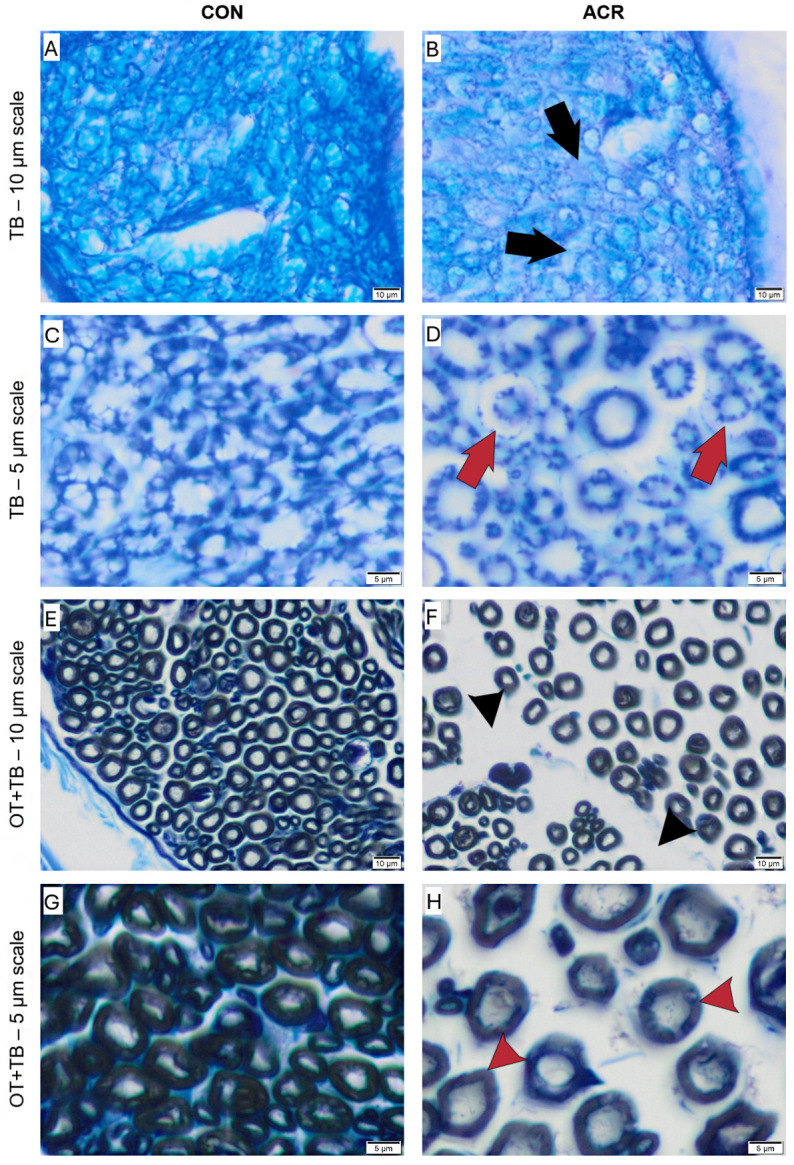
Representative toluidine blue and osmium tetroxide-fixed toluidine blue sections of rat sciatic nerve from the control (CON) and acrylamide-treated (ACR) groups. Panels (**A**–**D**) show toluidine blue-stained sections, and panels (**E**–**H**) show osmium tetroxide-fixed toluidine blue sections. Low-magnification images are shown in panels (**A**,**B**,**E**,**F**), whereas high-magnification images are shown in panels (**C**,**D**,**G**,**H**). Compared with the CON group, ACR-exposed nerves appeared to have less densely packed myelinated fiber profiles, more prominent inter-fiber spaces, and more irregular myelin sheath morphology, with qualitatively fewer large-diameter myelinated fibers. Arrows indicate representative pathological features: black arrows in (**B**) indicate endoneurial edema; red arrows in (**D**) indicate myelin degeneration/segmental degeneration; black arrowheads in (**F**) indicate axonal loss/advanced myelin degeneration; red arrowheads in (**H**) indicate thinned and abnormally shaped myelin. Scale bars: 10 µm in (**A**,**B**,**E**,**F**); 5 µm in (**C**,**D**,**G**,**H**).

**Figure 9:**
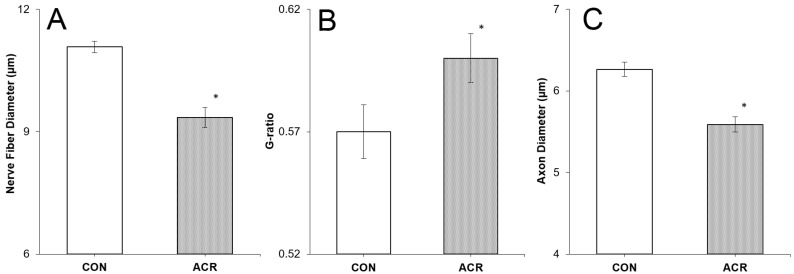
Quantitative morphometric analysis of sciatic nerves in the control (CON, n = 10) and acrylamide-treated (ACR, n = 9) groups. Bar graphs show mean ± SEM values for (**A**) nerve fiber diameter (µm), (**B**) G-ratio (axon diameter/fiber diameter), and (**C**) axon diameter (µm). * indicates a statistically significant difference between groups (*p* < 0.05).

**Table 1:** TOS (Total Oxidant Status), TAC (Total Antioxidant Capacity) values (mmol Trolox equivalent/L) and OSI (Oxidative Stress Index) values ((TOS/TAC) × 100) in both Serum and Tissue of experimental groups of animals. Data are expressed as mean ± SEM. * indicates a statistically significant difference between groups (*p* < 0.05).

	TOS (μmol H_2_O_2_ Equivalent/L)		TAC (mmol Trolox Equivalent/L)		OSI ((TOS/TAC) × 100)	
	Serum	Tissue	Serum	Tissue	Serum	Tissue
CON (n = 10)	4.79 ± 0.47	11.51 ± 0.46	1.53 ± 0.05	0.63 ± 0.05	315.42 ± 33.15	1925.72 ± 177.29
ACR (n = 9)	7.58 ± 1.17 *	17.36 ± 1.88 *	1.31 ± 0.13 *	0.31 ± 0.12 *	588.11 ± 81.22 *	5668.12 ± 669.15 *
*p* values	0.0117	0.0003	0.0116	0.0076	0.0140	0.0005

## Data Availability

The raw data supporting the conclusions of this article will be made available by the authors on request.
